# Relationships between lifestyle patterns and cardio-renal-metabolic parameters in patients with type 2 diabetes mellitus: A cross-sectional study

**DOI:** 10.1371/journal.pone.0173540

**Published:** 2017-03-08

**Authors:** Takeshi Ogihara, Tomoya Mita, Yusuke Osonoi, Takeshi Osonoi, Miyoko Saito, Atsuko Tamasawa, Shiho Nakayama, Yuki Someya, Hidenori Ishida, Masahiko Gosho, Akio Kanazawa, Hirotaka Watada

**Affiliations:** 1 Department of Metabolism & Endocrinology, Tokyo, Japan; 2 Center for Therapeutic Innovations in Diabetes, Tokyo, Japan; 3 Naka Memorial Clinic, Naka City, Ibaraki, Japan; 4 Department of Clinical Trial and Clinical Epidemiology, Faculty of Medicine, University of Tsukuba, Tsukuba, Ibaraki, Japan; 5 Center for Molecular Diabetology, Tokyo, Japan; 6 Sportology Center, Juntendo University Graduate School of Medicine, Tokyo, Japan; Shanghai Diabetes Institute, CHINA

## Abstract

**Introduction:**

While individuals tend to show accumulation of certain lifestyle patterns, the effect of such patterns in real daily life on cardio-renal—metabolic parameters remains largely unknown. This study aimed to assess clustering of lifestyle patterns and investigate the relationships between such patterns and cardio-renal-metabolic parameters.

**Participants and methods:**

The study participants were 726 Japanese type 2 diabetes mellitus (T2DM) outpatients free of history of cardiovascular diseases. The relationship between lifestyle patterns and cardio-renal-metabolic parameters was investigated by linear and logistic regression analyses.

**Results:**

Factor analysis identified three lifestyle patterns. Subjects characterized by evening type, poor sleep quality and depressive status (type 1 pattern) had high levels of HbA1c, alanine aminotransferase and albuminuria. Subjects characterized by high consumption of food, alcohol and cigarettes (type 2 pattern) had high levels of γ-glutamyl transpeptidase, triglycerides, HDL-cholesterol, blood pressure, and brachial-ankle pulse wave velocity. Subjects characterized by high physical activity (type 3 pattern) had low uric acid and mild elevation of alanine aminotransferase and aspartate aminotransferase. In multivariate regression analysis adjusted by age, gender and BMI, type 1 pattern was associated with higher HbA1c levels, systolic BP and brachial-ankle pulse wave velocity. Type 2 pattern was associated with higher HDL-cholesterol levels, triglycerides, aspartate aminotransferase, ɤ- glutamyl transpeptidase levels, and diastolic BP.

**Conclusions:**

The study identified three lifestyle patterns that were associated with distinct cardio-metabolic-renal parameters in T2DM patients.

**Trial registration:**

UMIN000010932

## Introduction

The incident of type 2 diabetes mellitus (T2DM) is related to numerous lifestyle problems. Furthermore, the incidence of cardiovascular disease (CVD) and diabetes related microvasuclar events is strongly related with lifestyle factors in patients with T2DM [1,2]. Lifestyle modifications such as a reduction in energy intake and an increase in physical activity can reduce the onset of T2DM [3] in non-T2DM population and the development of CVD in T2DM patients [4,5]. On the other hand, the reported effects of these interventions on the risks were very modest. Furthermore, a recent clinical trial that focused on the control of energy intake and increased physical activity in obese patients with T2DM showed no effect for lifestyle intervention on CVD [6]. In this regard, much attention should be paid to lifestyles other than diet and physical activity as lifestyle problems that could affect future CVD.

Recent studies have demonstrated the importance of sleep duration and/or quality as lifestyle factors, based on findings confirming the pathological roles of these factors in the onset of T2DM [7] and CVD [8]. Other studies reported that both sleep duration and/or quality can affect cardiovascular risk factors in T2DM patients [9–11].

Morningness-eveningness reflects the timing of the sleep-wake pattern and different aspects of sleep duration and/or quality, which are quite variable among individuals. In individuals of the evening type, social rhythms do not synchronize with the circadian clock. Previous studies demonstrated that evening type individuals tend to have unhealthy eating habits, behavioral health problems, and sleep complains more than morning type subjects [12,13]. More recently, we found inadequate glycemic control in evening type patients with T2DM [14] [15]. These findings suggest that evening type individuals potentially have impaired metabolism by abnormal circadian rhythm.

The prevalence of depression is reportedly higher in T2DM patients than non-T2DM [16]. Given that T2DM-related depression is related to poor glycemic control [17] and micro- and macro-vascular complications [18], partly due to increased counter-regulatory hormones [19], the depression status may be an important risk factor for defective glycemic control and T2DM-related complications in patients with T2DM.

In daily life, people have variable lifestyle patterns that can influence their cardio-renal-metabolic parameters. In this regard, it is conceivable that certain specific lifestyle patterns tend to accumulate in individual patients with T2DM. If we can classify the patients according to such accumulated lifestyle patterns, it would be useful to design treatment strategies tailored to the individual patients with T2DM. The aim of the present study was to explore possible classification of patients with T2DM free of history of CVD, according to clustering of lifestyle factors and mental status by factor analysis. We then investigated the relationships between each cluster of lifestyle factors (e.g., circadian rhythm, habitual sleep, mental state, diet, alcohol consumption, cigarettes consumption, physical activity) and cardio-renal—metabolic parameters, including brachial-ankle pulse wave velocity (baPWV), which is considered a useful predictor of CVD [20].

## Materials and methods

### Patients

The subjects of this cohort study were recruited from the Diabetes Outpatient Clinic of Juntendo University, (Tokyo, Japan) Naka Memorial Clinic (Naka, Japan) or Secomedic Hospital (Funabashi, Japan) as previously described [15] [21] [22]. T2DM patients who were between 25 and 70 years of age without history of apparent CVD were included.

A total of 1,032 consecutive subjects were screened as previously described [15]. Briefly, among them, 906 patients who met the above eligibility criteria were invited to participate in the present study. After providing information on the purpose and procedures of the study, 736 patients with T2DM were recruited. The study was approved by the Institutional Review Board of Juntendo University Hospital and conducted in accordance with the principles described in the Declaration of Helsinki. All patients provided written informed consent prior to participation. The study has been registered on the University Hospital Medical Information Network Clinical Trials Registry (UMIN000010932).

### Questionnaire survey

Questionnaire survey was conducted using valid and reliable self-administered questionnaires described previously [15]. Briefly, we used the Morning Evening Questionnaire (MEQ) [23], which is a self-assessment questionnaire developed primarily for screening candidates for sleep-related experiments to evaluate morningness and eveningness in individuals. A high MEQ score represents morning type.

The Pittsburg Sleep Quality Index (PSQI) [24] is a self-administered questionnaire designed to evaluate sleep quality and consists of 18 items that in turn are comprised of 7 components, with each weighted equally on a 0–3 scale to be summed to yield the global PSQI score ranging from 0 to 21, where the higher the scores, the worse the sleep quality.

The participating patients also completed the BDI (Beck Depression inventory)-II, which is a 21-item questionnaire [25]. A high BDI score represents depressive state.

Dietary habits during the preceding month were assessed with the validated, Brief, self-administered Diet History Questionnaire (BDHQ). The BDHQ is a 4-page structured questionnaire that asks about consumption frequency of selected foods to estimate the dietary intake of 56 food and beverage items with specified serving size described in terms of consumption in general Japanese populations[26].

Physical activity level was assessed with the International Physical Activity Questionnaire (IPAQ) that comprises four simple questions on physical activity [27]. The IPAQ results are expressed as metabolic equivalent scores (METs·hour-week^−1^).

Workers were defined as full-time employees or shift workers by a question in the questionnaire as described previously [15]. The subjects were also divided into non-smokers, former smokers or current smokers, as described previously[15]. Estimation of the total number of cigarettes was calculated by cigarettes smoked per day times smoking duration (years).

### Biochemical tests

Blood samples were obtained at visits after overnight fast. Liver and renal function tests, lipids, HbA1c, and glucose, (National Glycohemoglobin Standardization Program) were measured with standard techniques. Urinary albumin excretion (UAE) was measured by latex agglutination assay using a spot urine sample. The estimated glomerular filtration rate (eGFR) was calculated by the formula: eGFR (ml/min per 1.73 m^2^) = 194× Age^-0.287^× serum creatinine^-0.1094^ (×0.739 for females) [28].

### Measurement of baPWV

baPWV was measured using an automatic waveform analyzer (BP-203RPE; Colin Medical Technology, Komaki, Japan), as described previously [15] [29]. All scans were automatically conducted by well-trained investigators who were blinded to the clinical information.

### Statistical analysis

Results are presented as mean±SD or median (interquartile range: 25% to 75%) for continuous variables or number (proportion) of patients for categorical variables. Some parameters were logarithmically transformed to approximate a normal distribution. We used factor analysis with oblique promax rotation to reduce the complexity of lifestyle patterns of patients with T2DM by morningness-eveningness, quality of sleep including sleep duration, depressive status, energy intake, alcohol consumption, cigarettes smoking, and physical activity. Factors with an eigenvalue >1 were retained. Individual metabolites with a factor loading of >|0.3| are reported as composing that factor for simplicity. The factor scores for each lifestyle pattern and for each subject were calculated by summing each lifestyle score weighted by their factor loadings. The estimated factor scores were categorized into quintiles. Trend association across the quintile was evaluated by linear regression analysis for continuous variables and logistic regression analysis for categorical variables. We developed three models to evaluate the trend. The first model was unadjusted, second model was adjusted for age and gender, and third model adjusted for age, gender, and body mass index (BMI). The model for eGFR was only adjusted for BMI. Multivariate regression analysis adjusting for age, gender and BMI was performed to investigate what types of lifestyle patterns identified in this study were related with cardio-renal-metabolic parameters. Statistical tests were two-sided with 5% significant level. All analyses were performed using the SAS software version 9.3 (SAS Institute, Cary, North Carolina).

## Results

Ten patients were thus excluded from analysis because they did not complete the questionnaires among 736 patients. The characteristics of 726 subjects were shown in Table 1. The mean age was 57.8±8.6 years, 62.9% male and HbA1c was 7.0±1.0%. Glucose and lipid metabolisms and BP were well controlled in those subjects.

**Table 1:** Patients characteristics (n = 726).

Demographic data	
Age (years)	57.8±8.6
Gender (male)	456 (62.9)
Estimated duration of diabetes (years)	9.9±7.2
Body mass index (kg/m^2^)	24.6±4.1
HbA1c (%)	7.0±1.0
HbA1c (mmol/mol)	52.5±10.8
Fasting blood glucose (mg/dl)	134±31
Systolic blood pressure (mmHg)	127±14
Diastolic blood pressure (mmHg)	77±11
Total cholesterol (mg/dL)	185±28
HDL-cholesterol (mg/dL)	59±14
Triglyceride (mg/dL)	100 [70, 152]
AST (U/L)	21 [18, 27]
ALT (U/L)	22 [16, 33]
ɤ-GTP (U/L)	25 [17, 39]
Uric Acid (mg/dl)	5.5 ± 1.2
eGFR (ml/min/ 1.73 m^2^)	78 ± 18
UAE (mg/g creatinine)	10 [6, 23]
baPWV (cm/s)	1543± 279
Morningness-Eveningness Questionnaire	57.4 ± 7.3
Pittsburg Sleep Quality Index	5.1 ± 3.0
Beck Depression inventory -II	9.9 ± 7.6
Energy intake (kcal/day)	1713±582
Physical activity (Mets·h/week)	42.8±70.5
Sleep duration (hours)	6.4±1.2
Number of cigarettes	335±458
Current smoker (yes)	174 (24.0)
Alcohol (g/day)	12.3±21.5
On treatment for (n/%)	
Diabetes	620 (85.5)
Hypertension	346 (47.7)
Hyperlipidemia	442 (61.0)

Data are mean±SD or number (proportion) of patients. ALT: alanine aminotransferase; AST: aspartate aminotransferase; baPWV: brachial-ankle pulse wave velocity; eGFR: estimated glomerular filtration rate, HDL-C: high-density lipoprotein-cholesterol, UAE, urinary albumin excretion, ɤ-GTP, ɤ-glutamyl transpeptidase.

Factor analysis with oblique promax rotation identified three life style patterns (Table 2). Factor 1 seemed to be characterized by morningness-eveningness, sleep quality and depressive status (Type 1 pattern), Factor 2 by consumption of food, alcohol and cigarettes (Type 2 pattern), and Factor 3 by physical activity (Type 3 Pattern).

**Table 2:** Life style items and factor analysis with oblique promax rotation.

Components and item labels	Factor 1	Factor 2	Factor 3
Morningness Eveningness Questionnaire	**-0.62**	0.12	0.21
Pittsburg Sleep Quality Index	**0.78**	0.02	0.08
Beck Depression inventory	**0.71**	0.06	0.01
Energy intake	0.12	**0.64**	0.29
Smoking	-0.05	**0.65**	-0.22
Alcohol intake	-0.05	**0.81**	-0.04
International Physical Activity Questionnaire	-0.05	-0.06	**0.93**
Contribution	22%	21%	14%

The characteristics across quintile of lifestyle pattern on each type are shown in Tables 3–5. Table 3 shows the characteristics of the study subjects based on the quintiles of type 1 pattern scores. Subjects with a higher score for this pattern represent more evening type, poor sleep quality, and depressive status. They tended to be young, female, and worker or shift worker in the unadjusted model. Also, they tended to go to bed late, wake up late and sleep for shorter time. In addition, those patients tended to have less frequently have breakfast, and have late dinner, and late breakfast, frequently have late evening snacks, and consumption of large amount of food. These findings suggest they seem to consume greater percentage of their daily energy at late time. In addition, they has significantly higher BMI, alanine aminotransferase (ALT) levels, HbA1c levels and fasting blood glucose levels in the unadjusted model. Furthermore, fasting blood glucose and HbA1c levels were significantly higher in patients with higher scores of type 1 pattern even in the model adjusted for age, gender and BMI. Also, ALT and UAE were higher in the adjusted model by age and gender.

**Table 3:** Characteristics according to quintile categories of type 1 life style pattern score.

Variable	Quintile 1	Quintile 2	Quintile 3	Quintile 4	Quintile 5	Est1	Est2	Est3
MEQ	64.6 ± 4.9	60.2 ± 4.9	56.5 ± 5.7	54.4 ± 6.4	51.4 ± 6.4	-3.22¶	-2.96¶	-2.95¶
PSQI	2.6 ± 1.3	3.7 ± 1.4	4.6 ± 1.7	5.9 ± 2.1	8.7 ± 3.6	1.45¶	1.49¶	1.48¶
BDI	3.6 ± 3.6	6.6 ± 4.6	8.3 ± 4.8	11.9 ± 5.7	19.0 ± 8.1	3.61¶	3.72¶	3.72¶
Energy intake (kcal/day)	1609 ± 463	1709 ± 535	1650 ± 567	1811 ± 559	1782 ± 733	44.6†	68.7¶	68.6¶
Smoking (number)	388 ± 466	318 ± 453	357 ± 519	328 ± 460	279 ± 379	-20.8	12.5	11.9
Alcohol (g/day)	16.2 ± 23.2	11.8 ± 19.6	9.6 ± 21.6	13.0 ± 21.3	10.5 ± 21.3	-1.04	-0.01	-0.06
Physical activity (Mets·h/week)	47.5 ± 60.8	38.2 ± 56.4	45.7 ± 77.0	43.0 ± 81.6	40.1 ± 74.8	-1.00	-0.02	0.27
Age (years)	61.2 ± 6.3	59.8 ± 7.6	56.9 ± 8.8	56.1 ± 9.3	54.8 ± 9.0	-1.65¶	-	-
Gender (male)	107 (74.3)	95 (66.0)	83 (57.6)	93 (64.6)	73 (50.7)	-0.21¶	-	-
Body mass index (kg/m^2^)	23.9 ± 3.5	23.9 ± 3.7	24.8 ± 4.2	24.8 ± 3.8	25.8 ± 4.7	0.46¶	0.18	-
Estimated duration of diabetes (years)	10.3 ± 7.4	9.4 ± 6.3	8.6 ± 7.8	10.8 ± 7.6	10.4 ± 6.7	0.17	0.58	0.62
Anti-diabetes medications (yes)	123(85.4)	121(84.0)	121(84.0)	126(87.5)	125(86.8)	0.05	0.07	0.07
Anti-hypertensives (yes)	67(46.5)	72(50.0)	59(41.0)	73(50.7)	73(50.7)	0.04	0.08	0.06
Lipid-lowering agents (yes)	86(59.7)	90(62.5)	90(62.5)	86(59.7)	88(61.1)	0.00	0.01	0.00
Working (yes)	96(66.7)	101(70.1)	106(73.6)	110(76.4)	116(80.6)	0.18†	0.15*	0.15*
Shift worker (yes)	9(6.3)	16(11.1)	13(9.0)	19(13.2)	20(13.9)	0.18*	0.07	0.08
Sleep duration (hours)	7.1±1.1	6.7±0.9	6.4±1.0	6.2±1.2	5.9±1.2	0.07*	0.02	0.02
Wake time, A.M.	5:30[5:00,6:00]	6:00[5:18,6:30]	6:00[5:30,6:30]	6:00[5:30,6:30]	6:18[5:30,7:00]	-0.30¶	-0.27¶	-0.27¶
Bed time, P.M.	22:15[21:30,23:00]	23:00[22:00,23:30]	23:08[22:20,24:00]	23:15[22:30,24:00]	23:50[23:00,24:30]	0.19¶	0.18¶	0.18¶
Breakfast time, A.M.	7:00[6:15,7:18]	7:00[6:30,7:30]	7:00[6:30,7:30]	7:00[6:30,7:30]	7:20[6:40,8:00]	0.33¶	0.24¶	0.24¶
Dinner time, P.M.	19:00[18:30,19:30]	19:00[18:30,19:30]	19:00[19:00,20:00]	19:00[18:30,20:00]	19:30[19:00,20:00]	0.14¶	0.13¶	0.13¶
Number of breakfasts (/week)	6.9±0.6	6.8±0.8	6.7±0.9	6.5±1.4	6.0±1.9	0.18¶	0.14¶	0.13¶
Late evening snack (yes)	38 (26.4)	50 (34.7)	68 (47.2)	68 (47.2)	64 (44.4)	0.21¶	0.22¶	0.22¶
AST (U/L)	21 [18,27]	21 [18,27]	22 [17,25]	21 [18,26]	21 [18,28]	0.00	0.00	0.00
ALT (U/L)	21 [16,31]	23 [16,35]	22 [16,32]	24 [17,34]	24 [16,38]	0.04¶	0.03*	0.02
ɤ-GTP (U/L)	25 [17,36]	24 [17,35]	25 [18,40]	27 [18,46]	22 [15,42]	0.02	0.02	0.01
Uric Acid (mg/dl)	5.5 ± 1.2	5.5 ± 1.2	5.5 ± 1.2	5.5 ± 1.3	5.4 ± 1.3	-0.01	0.00	-0.01
eGFR (ml/min/ 1.73 m^2^)	76 ± 18	78 ± 18	79 ± 17	77 ± 18	80 ± 17	0.72	-	0.51
Total cholesterol (mg/dl)	186 ± 25	185 ± 31	186 ± 30	183 ± 26	187 ± 27	0.03	-0.45	-0.55
HDL-C (mg/dl)	62 ± 15	59 ± 15	59 ± 14	57 ± 14	59 ± 13	-0.59	-0.46	-0.30
Triglycerides (mg/dl)	94 [66,146]	98 [67,145]	96 [69, 139]	112 [77, 164]	104 [73, 152]	0.03*	0.02	0.01
Fasting blood glucose (mg/dl)	127 ± 28	134 ± 32	131 ± 27	136 ± 32	141 ± 34	2.90¶	2.29†	2.21*
HbA1c (%)	6.7 ± 0.8	6.9 ± 1.0	6.9 ± 1.0	7.1 ± 1.1	7.1 ± 1.1	0.11¶	0.07†	0.07*
HbA1c (mmol/mol)	49.5±8.3	52.4±10.8	52.2±10.5	54.0±12.0	54.6±11.6			
Systolic BP (mmHg)	127 ± 15	125 ± 14	125 ± 14	127 ± 13	129 ± 15	0.61	0.71	0.52
Diastolic BP (mmHg)	77 ± 13	76 ± 10	76 ± 10	78 ± 11	78 ± 12	0.36	0.08	-0.04
UAE (mg/g creatinine)	10 [6, 21]	9[6, 15]	10 [6, 24]	11 [6, 35]	11 [6, 26]	0.06	0.08*	0.07
baPWV (cm/s)	1582 ± 304	1563 ± 302	1514 ± 274	1540 ± 266	1517 ± 246	-15.31	9.56	9.90

Data are mean±SD, median [range: 25% to 75%] or number of subjects (proportion) before adjustment. Est1: Trend estimation for linear trends across quintiles is based on linear regression analysis for continuous variables or logistic regression analysis for categorical variables. Est2: Trend estimation for linear trends across quintiles is based on linear regression analysis for continuous variables or logistic regression analysis for categorical variables adjusted for age and gender. Est3: Trend estimation for linear trends across quintiles is based on linear regression analysis for continuous variables or logistic regression analysis for categorical variables adjusted for age, gender and BMI. eGFR is adjusted for BMI.

*P<0.05,

^†^P<0.01,

^¶^P<0.001 for testing the trend.

For trend test, log-transformed AST, ALT,ɤ-GPT, UAE, and TG. ALT: alanine aminotransferase; AST: aspartate aminotransferase; baPWV: brachial-ankle pulse wave velocity; BP: blood pressure; BDI: Beck Depression inventory, eGFR: estimated glomerular filtration rate, Est, estimation of regression coefficient, HDL-C: high-density lipoprotein-cholesterol, MEQ: morningness-eveningness questionnaire, PSQI: Pittsburg Sleep Quality Index, UAE, urinary albumin excretion,ɤ-GTP,ɤ-glutamyl transpeptidase.

**Table 4:** Characteristics according to quintile categories of type 2 pattern score.

Variable	Quintile 1	Quintile 2	Quintile 3	Quintile 4	Quintile 5	Est1	Est2	Est3
MEQ	55.3 ± 7.2	57.6 ± 7.0	57.0 ± 7.4	57.7 ± 6.4	59.6 ± 7.9	0.87†	0.62*	0.63*
PSQI	5.1 ± 3.4	5.1 ± 2.9	4.9 ± 2.5	5.6 ± 3.5	4.9 ± 2.9	0.00*	0.24*	0.23*
BDI	9.1 ± 7.9	10.6 ± 8.1	9.8 ± 7.4	9.6 ± 7.8	10.3 ± 7.2	0.12	0.75†	0.75†
Energy intake (kcal/day)	1144 ± 241	1531 ± 272	1711 ± 385	1913 ± 480	2262 ± 707	261.7¶	302.61¶	302.9¶
Smoking (number)	25 ± 79	88 ± 186	284 ± 314	446 ± 346	827 ± 615	196.3¶	168.3¶	168.1¶
Current smoker (yes)	11 (7.6)	19 (13.2)	39 (27.1)	51 (35.4)	50 (34.7)	0.45¶	0.38¶	0.39¶
Alcohol (g/day)	0.6 ± 2.1	0.8 ± 2.3	3.9 ± 6.5	12.4 ± 13.0	43.3 ± 28.1	9.71¶	9.62¶	9.61¶
Physical activity (Mets·h/week)	41.4 ± 68.1	38.5 ± 61.3	47.3 ± 68.9	40.2 ± 65.5	47.2 ± 87.1	1.31	0.37	0.60
Age (years)	56.7 ± 9.5	57.5 ± 8.5	57.3 ± 8.5	57.3 ± 8.7	59.9 ± 7.3	0.62†	-	-
Gender (male)	35 (24.3)	63 (43.8)	90 (62.5)	126 (87.5)	137 (95.1)	1.01¶	-	-
Body mass index (kg/m^2^)	24.6 ± 4.1	24.9 ± 4.0	24.4 ± 4.4	24.9 ± 4.1	24.4 ± 3.6	-0.04	0.14	-
Estimated duration of diabetes (years)	9.5±8.3	10.2±6.8	9.3±6.3	9.8±6.9	10.8±7.6	0.24	0.13	0.16
Anti-diabetes medications (yes)	127(88.2)	124(86.1)	119(82.6)	122(84.7)	124(86.1)	-0.04	-0.10	-0.09
Anti-hypertensives (yes)	68(47.2)	60(41.7)	56(38.9)	70(48.6)	90(62.5)	0.15†	0.17†	0.16*
Lipid-lowering agents (yes)	97(67.4)	96(66.7)	91(63.2)	83(57.6)	73(50.7)	-0.18†	-0.15*	-0.16*
Working (yes)	100(69.4)	90(62.5)	108(75.0)	117(81.3)	114(79.2)	0.20†	0.04	0.05
Shift worker (yes)	22(15.3)	13(9.0)	18(12.5)	19(13.2)	5(3.5)	-0.21*	-0.03	-0.02
Sleep duration (hours)	6.4±1.1	6.4±1.1	6.4±1.1	6.4±1.0	6.7±1.3	0.07*	0.01	0.01
Wake time, A.M.	6:00[5:30,7:00]	6:00[5:25,6:30]	6:00[5:30,6:30]	6:00[5:30,6:30]	6:00[5:00,6:30]	-0.11†	-0.15†	-0.16¶
Bed time, P.M.	23:00[22:30,24:00]	23:00[22:00,24:00]	23:00[22:30,23:50]	23:00[22:00,23:50]	22:30[21:30,23:30]	-0.12†	-0.04	-0.04
Breakfast time, A.M.	7:00[6:30,7:45]	7:00[6:30,7:40]	7:00[6:25,7:30]	7:00[6:30,7:30]	7:00[6:30,7:30]	-0.08†	-0.02	-0.02
Dinner time, P.M.	19:20[18:30,20:00]	19:00[18:30,19:30]	19:00[18:30,19:40]	19:00[18:30,20:00]	19:00[18:30,20:00]	0.00	-0.01	-0.01
Number of breakfasts (/week)	6.6±1.1	6.7±1.0	6.6±1.3	6.7±1.1	6.4±1.7	-0.05	-0.05	-0.04
Late evening snack (yes)	47(32.6)	64(44.4)	73(50.7)	60(41.7)	44(30.6)	-0.03	0.04	0.04
AST (U/L)	21 [18,26]	21 [18,26]	20 [17,25]	22 [17,28]	23 [20,29]	0.02*	0.02	0.02
ALT (U/L)	20 [15,31]	21 [16,34]	22 [16,29]	25 [17,35]	24 [17,34]	0.02	0.02	0.02
ɤ-GTP (U/L)	21 [15,32]	24 [16,35]	22 [15,36]	27 [20,44]	35 [22,57]	0.11¶	0.08¶	0.07¶
Uric Acid (mg/dl)	5.2 ± 1.2	5.3 ± 1.2	5.3 ± 1.2	5.8 ± 1.3	5.9 ± 1.1	0.20¶	0.04	0.03
eGFR (ml/min/ 1.73 m^2^)	79 ± 19	77 ± 18	80 ± 20	77 ± 16	77 ± 17	-0.42	-	-0.40
Total cholesterol (mg/dl)	187 ± 27	188 ± 28	185 ± 30	184 ± 28	182 ± 26	-1.29	0.81	0.73
HDL-C (mg/dl)	62 ± 13	59 ± 14	58 ± 15	57 ± 14	61 ± 14	-0.25	1.11†	1.24†
Triglycerides (mg/dl)	94 [67, 130]	99 [69, 146]	104[74, 139]	108 [72, 157]	117 [70, 165]	0.04†	0.05†	0.04*
Fasting blood glucose (mg/dl)	134 ± 33	135 ± 35	128 ± 23	134 ± 30	138 ± 32	0.84	1.04	0.98
HbA1c (%)	7.0 ± 1.1	7.1 ± 1.1	6.9 ± 0.8	7.0 ± 1.1	6.8 ± 0.9	-0.06*	-0.02	-0.03
HbA1c (mmol/mol)	53.5±11.7	53.9±12.1	51.7±8.7	52.8±11.5	50.8±9.5			
Systolic BP (mmHg)	128 ± 14	124 ± 14	124 ± 14	128 ± 15	129 ± 14	0.68	1.28†	1.13†
Diastolic BP (mmHg)	77 ± 9	74 ± 9	75 ± 11	79 ± 14	80 ± 10	1.11¶	0.83*	0.74*
UAE (mg/g creatinine)	11 [7, 25]	11 [6, 21]	8 [5, 15]	9 [5, 21]	13 [7, 35]	0.03	0.07	0.06
baPWV (cm/s)	1490 ± 245	1530 ± 281	1519 ± 271	1566 ± 272	1611 ± 313	278¶	16.9*	17.1*

Data are mean±SD, median [range: 25% to 75%] or number of subjects (proportion) before adjustment. Est1: Trend estimation for linear trends across quintiles is based on linear regression analysis for continuous variables or logistic regression analysis for categorical variables. Est2: Trend estimation for linear trends across quintiles is based on linear regression analysis for continuous variables or logistic regression analysis for categorical variables adjusted for age and gender. Est3: Trend estimation for linear trends across quintiles is based on linear regression analysis for continuous variables or logistic regression analysis for categorical variables adjusted for age, gender and BMI. eGFR is adjusted for BMI.

*P<0.05,

^†^P<0.01,

^¶^P<0.001 for testing the trend.

For trend test, log-transformed AST, ALT,ɤ-GPT, UAE, and TG. See Table 3 for abbreviations.

**Table 5:** Characteristics according to quintile categories of type 3 life style pattern score.

Variable	Quintile 1	Quintile 2	Quintile 3	Quintile 4	Quintile 5	Est	Est1	Est2
MEQ	53.6 ± 7.6	56.2 ± 6.2	58.5 ± 7.1	59.7 ± 6.7	59.1 ± 7.2	1.44¶	1.31¶	1.30¶
PSQI	4.4 ± 2.4	4.7 ± 2.6	5.4 ± 2.9	5.6 ± 3.2	5.5 ± 3.7	0.32¶	0.32¶	0.35¶
BDI	10.0 ± 7.5	10.0 ± 7.7	9.9 ± 7.3	9.0 ± 7.1	10.5 ± 8.7	0.00	0.03	0.04
Energy intake (kcal/day)	1354 ± 391	1538 ± 370	1680 ± 390	1968 ± 559	2022 ± 797	176.6¶	179.8¶	180.4¶
Smoking (number)	655 ± 636	300 ± 392	208 ± 329	255 ± 559	252 ± 323	-85.2¶	-85.8¶	-85.5¶
Alcohol (g/day)	8.9 ± 21.6	10.7 ± 19.6	11.0 ± 19.6	13.0 ± 23.9	13.6 ± 22.7	0.44	0.54	0.57
Physical activity (Mets·h/week)	8.9 ± 10.3	12.6 ± 11.9	19.2 ± 16.2	35.8 ± 24.4	138.1 ± 110.3	28.17¶	28.22¶	28.08¶
Age (years)	56.8 ± 8.4	56.9 ± 9.0	57.7 ± 8.1	58.6 ± 8.4	58.7 ± 8.9	0.56*	-	-
Gender male (%)	102 (70.8)	80 (55.6)	91 (63.2)	86 (61.1)	90 (62.5)	-0.05	-	-
Body mass index (kg/m^2^)	25.1 ± 4.2	24.6 ± 4.2	24.8 ± 4.1	24.8 ± 4.2	24.0 ± 3.6	-0.21*	-0.12	-
Estimated duration of diabetes (years)	9.4±6.1	10.1±7.2	9.4±7.0	10.3±8.0	10.5±7.6	0.24	0.12	0.10
Anti-diabetes medications (yes)	127(88.2)	125(86.8)	120(83.3)	118(81.9)	126(87.5)	-0.05	-0.05	-0.05
Anti-hypertensives (yes)	78(54.2)	67(46.5)	67(46.5)	77(53.5)	55(38.2)	-0.10	-0.11*	-0.10
Lipid-lowering medications (yes)	87(60.4)	87(60.4)	91(63.2)	88(61.1)	87(60.4)	0.00	-0.01	-0.01
Working (yes)	113(78.5)	107(74.3)	98(68.1)	102(70.8)	109(75.7)	-0.05	0.04	0.04
Shift worker (yes)	15(10.4)	10(6.9)	13(9.0)	18(12.5)	21(14.6)	0.15	0.18*	0.18*
Sleep duration (hours)	6.6±1.2	6.6±1.1	6.4±1.1	6.3±1.2	6.4±1.3	-0.07*	-0.08†	-0.08†
Wake time, A.M.	6:00[5:30,6:50]	6:00[5:30,6:30]	6:00[5:13,6:30]	6:00[5:30,6:30]	5:55[5:00,6:23]	-0.10†	-0.10*	-0.09*
Bed time, P.M.	23:15[22:30,24:20]	23:00[22:20,24:00]	23:00[22:08,23:45]	23:00[22:00,23:50]	22:30[22:00,23:30]	-0.16¶	-0.13¶	-0.13¶
Breakfast time, A.M.	7:00[6:30,8:00]	7:00[6:30,7:30]	7:00[6:30,7:30]	7:00[6:30,7:30]	7:00[6:25,7:30]	-0.04	-0.05	-0.04
Dinner time, P.M.	19:30[19:00,20:00]	19:00[18:30,20:00]	19:00[18:30,19:30]	19:00[18:30,20:00]	19:00[18:30,19:30]	-0.08†	-0.06*	-0.06*
Number of breakfasts (/week)	6.2±1.8	6.7±0.8	6.7±1.1	6.7±1.1	6.6±1.3	0.08*	0.06	0.06
Late evening snack (yes)	60(41.7)	59(41.0)	53(36.8)	58(40.3)	58(40.3)	-0.01	-0.02	-0.02
AST (U/L)	20 [17,26]	20 [17,27]	21 [18,26]	22 [19,27]	23 [19,27]	0.03†	0.03†	0.03†
ALT (U/L)	21 [16,33]	21 [16,29]	24 [17,35]	23 [17,34]	24 [16,33]	0.02	0.03*	0.04†
ɤ-GTP (U/L)	26 [18,43]	26 [17,38]	26 [17,43]	25 [18,36]	25 [16,38]	-0.03	-0.02	-0.01
Uric Acid (mg/dl)	5.8 ± 1.2	5.3 ± 1.3	5.5 ± 1.3	5.5 ± 1.2	5.3 ± 1.1	-0.09†	-0.07*	-0.06*
eGFR (ml/min/ 1.73 m^2^)	79 ± 18	79 ± 17	78 ± 19	76 ± 16	78 ± 18	-0.48	-	0.37
Total cholesterol (mg/dl)	182 ± 28	187 ± 27	186 ± 27	187 ± 29	184 ± 29	0.48	0.35	0.41
HDL-C (mg/dl)	57 ± 14	60 ± 13	57 ± 14	60 ± 13	62 ± 15	0.87¶	0.63	0.53
Triglycerides (mg/dl)	102 [81, 157]	97 [67, 162]	105 [71, 153]	100 [67, 145]	97 [66, 149]	-0.03*	-0.03	-0.02
Fasting blood glucose (mg/dl)	133 ± 32	135 ± 33	137 ± 32	131 ± 29	133 ± 29	-0.48	-0.13	-0.08
HbA1c (%)	6.9 ± 1.0	7.0 ± 1.0	7.0 ± 1.0	6.9 ± 1.0	7.0 ± 0.9	0.00	0.01	0.01
HbA1c (mmol/mol)	52.0±11.0	53.2±11.3	52.7±11.2	52.1±11.0	52.6±9.7			
Systolic BP (mmHg)	127 ± 15	127 ± 15	125 ± 13	127 ± 13	126 ± 15	-0.23	-0.28	-0.15
Diastolic BP (mmHg)	79 ± 10	78 ± 11	77 ± 11	77 ± 13	75 ± 11	-0.72*	-0.52	-0.45
UAE (mg/g creatinine)	10 [6, 24]	10 [6, 22]	10 [5, 25]	10 [6, 21]	10 [6, 21]	-0.03	-0.03	-0.02
baPWV (cm/s)	1568 ± 317	1523 ± 278	1532 ± 280	1539 ± 266	1554 ± 255	-1.22	-8.18	-8.39

Data are mean±SD, median [range: 25% to 75%] or number of subjects (proportion) before adjustment. Est1: Trend estimation for linear trends across quintiles is based on linear regression analysis for continuous variables or logistic regression analysis for categorical variables. Est2: Trend estimation for linear trends across quintiles is based on linear regression analysis for continuous variables or logistic regression analysis for categorical variables adjusted for age and gender. Est3: Trend estimation for linear trends across quintiles is based on linear regression analysis for continuous variables or logistic regression analysis for categorical variables adjusted for age, gender and BMI. eGFR is adjusted for BMI.

*P<0.05,

^†^P<0.01,

^¶^P<0.001 for testing the trend.

For trend test, log-transformed AST, ALT,ɤ-GTP, UAE, and TG. See Table 3 for abbreviations.

Table 4 lists the characteristics of the study subjects based on the quintiles of type 2 pattern scores. Subjects with a higher score of this pattern tended to be old, males, current smokers, and workers or shift workers in the unadjusted model. Furthermore, these patients tended to go to bed early, wake up early and eat breakfast early. Accordingly, they tended to be morning type, but less frequently had breakfast. With regard to the cardio-metabolic parameters, patients with higher score of type 2 pattern showed significantly higher aspartate aminotransferase (AST) levels, ɤ-glutamyl transpeptidase (ɤ-GTP) levels, uric acid and triglycerides. These data reflect patterns of alcoholic liver dysfunction, while there was no significant difference in BMI among the groups. On the other hand, HbA1c levels were significantly lower in these patients in the unadjusted model, but this finding was not observed in the adjusted model. The use of lipid-lowering medications, especially statin but not fibrate (data not shown), was lower in patients with a higher score of type 2 pattern. With regard to BP, these patients had high systolic and diastolic BP despite high use of antihypertensive drugs. Also, baPWV was significantly higher in those patients. According to the adjusted models by age and gender and by age, gender, and BMI, subjects with a higher score for this pattern had higher ɤ-GTP, triglyceride, HDL-cholesterol levels, systolic and diastolic BP, and baPWV.

Table 5 shows the characteristics of the study subjects according to the quintiles of type 3 pattern scores. Subjects with a higher score for this pattern were old, non-smokers, shift workers, and morning type. These patients are likely to have low BMI despite the large amount of food intake. They tended to go to bed early, wake up early, sleep for short duration and have early dinner. Patients with a higher score of type 3 pattern showed significantly lower uric acid, triglyceride and diastolic BP, and higher HDL-cholesterol. However, these subjects only had lower uric acid levels, rather higher AST and ALT, in the adjusted models by age and gender and by age, gender and BMI. They also had higher AST and ALT, and were less likely to use antihypertensive medication, in the adjusted model by age and gender.

Next, we performed a multivariate regression analysis to investigate whether three types of lifestyle patterns identified in this study were related with cardio-renal-metabolic parameters (Table 6). Type 1 pattern was associated with higher HbA1c levels, systolic BP and baPWV. Type 2 pattern was associated with higher HDL-cholesterol levels, triglycerides, AST, ɤ-GTP levels, and diastolic BP.

**Table 6:** Multivariate regression analysis adjusted for age, gender and BMI.

Variable	Lifestyle pattern	Standrized regression coefficient	P value
AST (U/L) (logarithmic transformation)	type 1	-0.15	0.88
	type 2	2.37	0.018
	type 3	1.63	0.1
ALT (U/L) (log) (logarithmic transformation)	type 1	1.45	0.15
	type 2	-0.17	0.86
	type 3	1.91	0.057
ɤ-GTP (U/L) (logarithmic transformation)	type 1	0.16	0.88
	type 2	5.30	<0.001
	type 3	-1.09	0.28
Uric Acid (mg/dl)	type 1	0.15	0.88
	type 2	1.32	0.19
	type 3	-1.48	0.14
Total cholesterol (mg/dl)	type 1	-0.23	0.82
	type 2	0.17	0.86
	type 3	0.98	0.33
HDL-C (mg/dl)	type 1	-0.31	0.75
	type 2	3.71	<0.001
	type 3	0.86	0.39
Triglycerides (mg/dl) (logarithmic transformation)	type 1	0.90	0.37
	type 2	1.98	0.048
	type 3	-0.78	0.44
HbA1c (%)	type 1	2.39	0.017
	type 2	-1.13	0.26
	type 3	0.77	0.44
Systolic BP (mmHg)	type 1	2.17	0.03
	type 2	1.72	0.086
	type 3	-0.10	0.92
Diastolic BP (mmHg)	type 1	0.73	0.47
	type 2	2.22	0.027
	type 3	-0.82	0.41
UAE (mg/g creatinine) (logarithmic transformation)	type 1	0.87	0.38
	type 2	1.44	0.15
	type 3	0.26	0.79
baPWV (cm/s)	type 1	1.99	0.047
	type 2	0.59	0.55
	type 3	-1.09	0.28

See Table 3 for abbreviations.

## Discussion

This is the first report on the relationship between clustered lifestyle, not a single aspect of lifestyle, and cardio-renal-metabolic parameters in patients with T2DM. Using factor analysis, lifestyle patterns in real daily life can be classified into three factors; morningness-eveningness, sleep quality and depressive state (type 1 pattern), consumption of food, alcohol and cigarettes (type 2 pattern), and physical activity (type 3 pattern). Interestingly, each lifestyle is associated with distinct cardio-renal -metabolic parameters.

Subjects with a higher score of type 1 pattern characterized by evening type, poor sleep quality and depressive status showed poor glycemic control and higher ALT and UAE levels in this study. It may be reasonable that these characteristics correlated well with each other because previous reports showed a tendency for such association[14] [15]. These individuals tend to have later dinners, frequent late evening snacks, and less frequent breakfast and consume more food. They were considered to consume a greater amount of their daily energy intake at late time of the day. A previous study demonstrated that late dinnertime increases in postprandial glucose levels after breakfast in the following morning compared to usual dinner time condition through a higher effect of late dinners on carbohydrate digestion and absorption of dietary carbohydrates [30]. Therefore, it seems that late eating could result in worsening of glycaemic control. In addition, consistent with our findings, evening type was shown to be related to more depressive symptoms [31]. Depressive status may also negatively affect glucose metabolism through increased counter-regulatory hormones [19].

Subjects with a higher score of type 1 pattern were more likely to be employed as workers, with higher frequency of overtime work beyond 21:00 PM (data not shown). Such workers may be often forced to stay awake through social cues against their preference. This may lead to disruption of the circadian system. A previous study demonstrated that forced circadian misalignment developed insulin resistance [32]. Therefore, circadian misalignment by environmental elements may contribute to worsening of glycemic control in those patients.

Patients with T2DM are known to have sleep abnormalities compared with healthy subjects [33]. In the present study, subjects with a higher score of type 1 pattern had poor sleep quality and short sleep duration. Recent studies reported both sleep quality and/or duration have negative influence on glucose and lipid metabolism in T2DM patients [9–11]. Sleep has major regulatory effects on metabolic function, hormone release and sympathovagal activity. Indeed, sleep deprivation is reported to be associated with low levels of circulating satiety hormone leptin, high levels of appetite stimulating hormone ghrelin [34] and high sympathetic activity [35]. These changes are expected to be deleterious for glycaemic control. Taken together, our findings suggest the synergistic deleterious effects of these unfavourable traits on glycemic control in patients with T2DM. In fact, our multivariate regression analysis suggested that type 1 pattern was associated with higher HbA1c levels even considering other lifestyle patterns. Thus, optimizing these unfavourable habits should be investigated as an intervention to improve glucose control in patients with T2DM.

Subjects with a higher score of type 1 pattern had high UAE levels. The high levels of UAE could be due to poor glycemic control and potentially high sympathetic activity. Due to the presence of accumulated risk factors for atherosclerosis, type 1 pattern was associated with increased atrial stiffness in the multivariate regression model.

Subjects with a high score of type 2 pattern reported high consumption of food, alcohol and cigarettes. Especially, they had high ɤ-GTP, uric acid, and triglyceride levels. These findings suggest the presence of alcohol-related metabolic dysfunctions. Also, the results of the multivariate regression analysis supported these findings. It is also plausible that the high BP found in these patients was due to the high consumption of alcohol and cigarette [36].

In general, subjects preferring eveningness have been shown to make a custom of adverse health behaviors such as smoking, alcohol use, and physical inactivity compared to those preferring morningness in the general population [37]. In contrast, subjects with a high score of type 2 pattern showed morning type despite having unfavourable habits such as high consumption of alcohol and cigarettes. The exact reason(s) for this discrepancy is (are) not clear at present. However, short sleep duration was reported to be associated with high alcohol consumption [38]. In this study, subjects with a high score of this pattern reported short sleep duration. The latter could adversely affect the classification of morningness-eveningness.

Our study also identified a unique clustered lifestyle characterized by physical activity. The results of multivariate adjusted model demonstrated no correlation with the beneficial cardio-renal-metabolic parameters in subjects with a high score of this pattern, except uric acid. Rather, these subjects had high AST and ALT levels. This is possibly because higher consumption of food could cancel the beneficial effects of habitual physical activity on cardio-renal metabolic parameters. These data suggest the presence of antagonistic effect of lifestyle patterns that tend to accumulate in real daily life on cardio-renal-metabolic parameters.

The present study has certain limitation. First, the cross-sectional design does not allow inference of causal relationship between lifestyle patterns and cardio-renal-metabolic parameters. Also, we could not exclude the possibility that subjects change their daily lifestyles at other periods. Second, we did not confirm the validity and reproducibility of the lifestyle patterns found in this study in a different Japanese population. In this regard, we assessed the validity and reproducibility of the lifestyle patterns in this study by the following steps: 1) Subjects data in this study were randomly divided into two datasets (test and validation groups) in half, 2) The lifestyle patterns were evaluated by factor analysis in each dataset, and 3) Steps 1) and 2) were repeated ten times. According to this analysis, the lifestyle patterns evaluated by factor analysis in test and validation groups were similar, and the results were almost identical to those in the whole population (data not shown), suggesting that validity and reproducibility of the lifestyle patterns were relatively high in an internal. Finally, there may be other lifestyle patterns that should be considered, although based on the results of previous reports, we chose the possible lifestyle factors related to cardio-renal—metabolic parameters such as sleep quality [10,39], morningness-eveningness trait [14,40,41], depression status [17,18], energy intake and physical activity [3–5], smoking [42] and alcohol consumption [43].

## Conclusions

The present study identified three clustering of lifestyle patterns and confirmed the relationship between such patterns and cardio-renal-metabolic parameters in T2DM patients free of history of CVD. The results highlight the importance of intervention to modifiable lifestyles based on their complex lifestyle pattern in daily life, in order to achieve appropriate cardio-renal-metabolic functions and prevent future CVD in patients with T2DM.

## Abbreviations

ALT: alanine aminotransferase
AST: aspartate aminotransferase
BaPWV: brachial-ankle pulse wave velocity
BDHQ: brief, self-administered diet history questionnaire
BDI: Beck Depression inventory
BMI: body mass index
BP: blood pressure
CVD: cardiovascular disease
eGFR: estimated glomerular filtration rate
ɤ-GTP: ɤ-glutamyl transpeptidase
HDL: high-density lipoprotein-cholesterol
IPAQ: International Physical Activity Questionnaire
MEQ: morningness-eveningness questionnaire
NGSP: National Glycohemoglobin Standardization Program
PSQI: Pittsburg Sleep Quality Index
T2DM: Type 2 diabetes mellitus
UAE: Urinary albumin excretion
